# Postnatal care and acceptability of emollient therapy in very low birthweight infants in Harare, Zimbabwe: a qualitative analysis

**DOI:** 10.1186/s12887-024-04661-x

**Published:** 2024-03-16

**Authors:** Alexa Hui, Gwendoline Chimhini, Margaret Saungweme, Dorah Kaisi, Elisabeth Munetsi, Hilda A. Mujuru, Gary L. Darmstadt

**Affiliations:** 1 Human Biology Program, Stanford University, Stanford, CA USA; 2 Child and Adolescent Health Unit, Faculty of Medicine and Health Sciences, University of Zimbabwe, Harare, Zimbabwe; 3 Prematurity Research Center, Department of Pediatrics, Stanford University School of Medicine, Stanford, CA USA

**Keywords:** Neonate, Preterm, Emollient, Skincare, Low birthweight, Skin barrier

## Abstract

**Background:**

Preterm birth (birth before 37 completed weeks of pregnancy) is the leading cause of neonatal and child under-five mortality globally, both of which are highest regionally in sub-Saharan Africa. The skin barrier plays a critical role in neonatal health and increasing evidence supports the use of topical emollient therapy to promote postnatal growth and reduce hospital-acquired infections in preterm infants. The World Health Organization (WHO) currently recommends emollient therapy in preterm or low birthweight infants globally but calls for further research on impacts of emollient use, especially in Africa. Little is known about postnatal skincare practices and the tradition of oil massage across sub-Saharan Africa. Further documentation is necessary to understand the context for future emollient intervention trials.

**Methods:**

61 semi-structured interviews with mothers who just delivered preterm or term infants and 4 focus group discussions (32 participants) with physician and nurse providers of newborn care were conducted at Sally Mugabe Central Hospital (SMCH), in Harare, Zimbabwe. SMCH is the principal public-sector tertiary care hospital for newborn infants in the northern part of the country. Mothers and healthcare professionals were questioned about newborn care at the hospital, current neonatal skincare and bathing practices, and the community’s receptivity to a future emollient therapy clinical trial.

**Results:**

Postnatal skincare is centrally important to Zimbabwean communities and petroleum jelly application is nearly universal. The use of cooking oil and other natural oils on infants is also part of traditional customs. The primary needs and desires of mothers who have just given birth to preterm infants are having greater agency in their children’s care and financial support in purchasing prescribed medications while at the hospital. Community receptivity to emollient therapy as a cost-effective treatment is high, particularly if mothers are trained to assist with the intervention.

**Conclusion:**

Emollient therapy will likely be well-received by communities in and around Harare because of its accordance with current skincare practices and perceptions; however, cultural norms and the experiences of new mothers who have given birth at a facility highlight challenges and considerations for future clinical trial execution.

**Trial registration:**

Clinicaltrials.gov NCT05461404.

## Background

Neonatal mortality currently accounts for almost half (47%) of all under-five child deaths globally [1]. Further, the average neonatal mortality rate (NMR) hides gross disparities within and across countries and regions. Neonatal mortality in sub-Saharan Africa (SSA) is the highest regionally in the world at 27 deaths per 1,000 live births, accounting for 42% of global newborn deaths [1]. At its current rate of decline, the NMR in SSA will not reach the Sustainable Development Goal target of 12 deaths per 1000 live births by 2030 [2]. 

The top cause of deaths in children before age 5 years is complications of preterm birth (birth before 37 completed weeks of pregnancy) [3]. Preterm and very low birthweight (VLBW, < 1500 g) infants who survive these early complications are at a higher risk for a lifetime of adverse health and development outcomes such as chronic pulmonary, cardiovascular, and metabolic disease; deficits in growth, hearing, vision, cognition, and other domains of development; behavioral problems, learning difficulties, and poor academic performance [4–6]. 

Skin barrier integrity is a critical factor influencing neonatal survival and health, particularly among preterm, VLBW infants [7]. As the interface between an infant’s internal and external environments, a developmentally mature skin barrier performs many essential functions; in contrast, the developmentally immature skin barrier of the preterm infant is functionally compromised, resulting in increased heat, water, and energy loss, and risk for invasion of pathogens through the skin [7]. These processes contribute to impairment of growth and neurodevelopment and increased risk for mortality [7]. In low-resource settings, where maternal malnutrition and poor adherence to infection prevention and control practices are more prevalent, risk is further magnified [8]. 

A growing body of literature supports the use of emollient therapy—massaging newborn infants with applications of topical preparations, particularly natural vegetable oils, which protect, moisturize, lubricate and soften the skin—in low- and middle-income countries (LMICs) to improve skin barrier function, promote weight gain and reduce rates of nosocomial infections, such as sepsis, and associated mortality in preterm or very low birthweight infants [9–15]. In 2022, the World Health Organization recommended that emollient therapy be considered in the care of preterm or low birthweight (LBW) infants globally, but also noted the need for further research on emollient use, especially in Africa [16, 17]. 

While application of oils and other products to the skin of newborn infants is a widespread practice throughout South Asia [18–20], there is relatively little documented evidence of this behavior in Africa [21]. Low-cost, community-accessible interventions such as emollient therapy can be an important tool in closing the gap in the quality of care for neonates and small and/or sick infants in LMICs.

This study examines the perceptions and practices of newborn skincare and bathing among mothers who recently gave birth at Sally Mugabe Central Hospital (SMCH) in Harare, Zimbabwe—the primary public-sector tertiary care hospital for newborn infants in the northern part of the country. Semi-structured interviews and focus group discussions (FGDs) provide insight into newborn skincare and bathing practices at healthcare facilities and at home, as well as mothers’ beliefs, perceptions, and opinions about such care. This work aims to address a research gap in the neonatal skincare traditions of countries in Southern Africa, with a focus on the urban population of Zimbabwe’s capital city, Harare, which accounts for approximately 16% of the country’s total population [22]. Further, the voices of mothers and healthcare professionals collected here provide important considerations for the operation of a future emollient therapy clinical trial at SMCH and the community’s receptivity of the trial.

## Methods

### Study setting

The patient population of SMCH comes from urban and suburban Harare and surrounding rural areas and approximates a population-representative sample. Mothers with pregnancy complications, including impending preterm birth, are referred to SMCH, primarily from the northern half of the country, for high-risk antenatal care and childbirth services. Mothers giving birth and children under five years old are treated for free at SMCH; however, parents are often expected to pay external health care providers for medicines and laboratory tests not available at the hospital. Approximately 12,000 births take place annually at SMCH. Most babies are delivered through normal vertex delivery (86%), 13% by cesarean section and 0.25% by vacuum-assisted vertex delivery. The stillbirth rate is 33 per 1000 total births whilst the early neonatal mortality rate (in the first week after birth) is 22 per 1000 live births. The top four causes of neonatal mortality are complications of prematurity, hypoxic ischemic encephalopathy, sepsis and gastroschisis. Mortality among VLBW infants is 54% [23]. 

Post-delivery mothers and their newborn infants are cared for in different wards depending on the mode of delivery; women who gave birth by normal vertex delivery are in Postnatal Ward A, and those who delivered by caesarean section are in Postnatal Ward B.

The SMCH neonatal unit (NNU) houses the country’s largest neonatal surgical unit which receives referrals from the northern half of the country. The 100-bed NNU includes a transition ward, also referred to as the preterm ward, comprised of 20 incubators for infants who weigh < 1.5 kg (kg) at birth, a 4 bedded Neonatal Intensive Care Unit, 20 bed neonatal surgical care unit, an observation ward and a 12-bed Kangaroo Mother Care (KMC) ward where infants who weigh $\ge$1.5 kg are nursed skin to skin on their mothers’ chests continuously and discharged when sufficient growth (e.g., >1.8 kg), ability to feed, and clinical stability have been achieved.

### Study design

Qualitative data was collected from March to May 2023 through 61 semi-structured interviews with mothers who had just delivered at SMCH, and four focus groups discussions (FGDs), two with nurses and two with doctors who worked at SMCH. This qualitative study did not include the application of any oil, plant, or medicinal product to participants’ skin.

### Interviews with mothers

Three data tools were developed to capture information about skincare practices and perceptions and were administered to an intended sample size of 20 respondents needed to reach saturation of information (Table 1). Respondents for Caregiver Survey #1 (CS1) focused on newborn skincare and bathing after birth in the hospital and Caregiver Survey #2 (CS2) on newborn skincare and emollient use at home after discharge of mothers who had given birth to full-term appropriate for gestational age infants. Equal numbers of mothers were recruited for both surveys from Postnatal Ward A and Postnatal Ward B. Both questionnaires focused on the skincare and bathing practices which newly delivered mothers performed on their most recent previous child, and featured questions about mothers’ beliefs and perceptions about the care their children received. All interviews took place at the respondent’s bedside for a duration of about 20 min (not including informed consent process).

**Table 1:** Recruitment criteria

Data Tool	Relevant Topics	Patients Recruited From	Patient Parity
**CS1**	First bath; skincare and bathing practices at the hospital/clinic	10 from Postnatal Ward A (normal vaginal delivery) 10 from Postnatal Ward B (post Cesarean delivery)	> 1
**CS2**	Skincare and oil use at home	10 from Postnatal Ward A (normal vaginal delivery) 10 from Postnatal Ward B (post Cesarean delivery)	> 1
**CS3**	Preterm infant delivery experience; care in the neonatal unit (NNU); perceptions of emollient therapy and participation in a future clinical trial	Kangaroo Mother Care Ward (infants ≥ 1.5 kg) Transit B (preterm) Ward (infants < 1.5 kg)	*10 first-time mothers (parity = 1), 10 mothers with more than one child (parity > 1)

*Note* One mother delivered twins during her first time, so even though her parity was > 1, her interview was included with that of first-time mothers

Respondents for Caregiver Survey #3 (CS3) were recruited from the NNU, where infants who are born preterm and/or LBW remain at the hospital until they meet criteria for discharge. Their mothers remain with them continuously (if in the KMC ward) or stay in a hostel attached to the hospital to feed their infants on a regular schedule. Half of the interviews were conducted with mothers from the KMC ward, whose infants have reached a birthweight ≥ 1.5 kg. The other half were conducted with mothers of children admitted to the preterm ward, whose infants are < 1.5 kg and under the constant care of nurses and doctors. Half of CS3 respondents were first-time mothers, and the other half had cared for a newborn child previously. Questions focused on the care of their current child (the one they just delivered), their receptivity to a clinical trial, and the future introduction of emollient therapy into their communities. Interviews were either conducted at the bedside (for mothers practicing kangaroo care) or in the tearoom in the NNU and did not exceed 40 min (not including informed consent process).

During recruitment, participants were approached at the bedside or in the ward and the purpose and procedures of the study explained. If they agreed to participate, they were then given a more in-depth explanation of the research and asked to sign a consent form as part of the informed consent process, during which they were given the opportunity to ask questions and to opt out. Only four potential individual interview respondents declined participation after being approached. One CS2 respondent consented to the interview but declined audio recording; per study protocol, notes from her interview were included in analysis, but she was not counted towards the overall target sample size of 60. To avoid contamination between individual interview respondents when interviews occurred at the bedside, only one type of each survey was conducted per cubicle per day in Postnatal Ward A, where mothers were discharged the same day or early the following day. In Postnatal Ward B, where mothers stayed several days, one type of each survey was conducted per cubicle per day, and the research team ensured that no cubicle was subsequently revisited for two consecutive days. Interviews were conducted either in Shona (local vernacular language) or English according to a respondent’s preference.

### Focus group discussions with healthcare providers

Two FGDs each were conducted with nurses and doctors separately. For each FGD, the research team reached out to one doctor or nurse, who then helped recruit the other participants based on shift scheduling and availability. Focus group target sizes were initially 5–8 individuals, to reach a minimum sample size of 20 participants; however, eight participants attended each FGD for a total sample of 32 healthcare professionals. All FGDs were conducted in English.

### Qualitative data collection and analysis

Both interviews and FGDs were audiotaped using a digital audio-recorder and complemented with written interview notes on paper. Demographic data were crosschecked by the research team for completeness and accuracy daily. During both English interviews and FGDs, research nurses occasionally helped translate between Shona and English for clarification. Interviews which occurred in Shona (77%) were translated into English by the research team. Qualitative coding was performed on the English transcriptions. A small sample (10%) of each interview was coded by a social scientist in Shona. Coding and analysis were compared line-by-line with the research team’s analysis of the corresponding English translations. A lack of discrepancies between the two ensured the fidelity of the research team’s interview translations.

Content analysis through a combination of inductive and deductive coding was performed on all interviews and FGDs based on emerging themes relevant to the research objectives. Coding of data was carried out using QSR Nvivo 14©, a computer program for analyzing qualitative datasets. Prior to the first round of coding, three “mini-codebooks” were created for the three separate data tools, each consisting of approximately 10–12 codes derived directly from questions in each survey. Two to three rounds of iterative coding were then performed on interviews within each data tool, during which codes were either consolidated into more general codes or split into more granular sub-codes. Next, new codes were created for topics not already included in the three mini-codebooks, but which emerged inductively across all interviews and FGDs. Finally, major themes were created from codes which overlapped most across all data types and which were relevant to neonatal skincare, maternal and infant wellbeing, and the community’s receptivity to an emollient therapy clinical trial. FGDs were then coded according to the established codebook to add supplemental information within each of these major themes. Dissemination of the study findings will be done post-publication, including discussion of the findings with the doctors and nurses still working at the hospital.

## Results

### Summary of respondents

Demographic information for the 61 respondents is summarized in Table 2. The mean and median age of respondents was 31 years, and the average number of children each mother had was approximately 3; 14 (23%) of the mothers previously gave birth to a preterm or VLBW infant. Almost all respondents reported that they lived in urban areas and completed secondary education. Most nurses who participated were specialists in midwifery; a few were general nurses (Table 3). Doctors were resident medical officers (interns) in Paediatrics, Obstetrics and Gynaecology, or Internal Medicine. Nurse focus groups represented a diversity of experience, ranging from 2 to 42 years. All doctors who participated had been practicing for 2 years or less.

**Table 2:** Summary of respondent demographics

	CS1	CS2	CS3
**Total # of Participants**	20	21	20
**Age**	**Frequency**		
19–24	0	2	7
25–29	3	3	6
30–34	8	10	4
35–39	8	5	3
40–44	1	1	0
**Location of Residence**	**Frequency**		
Urban	20	20	19
Peri-urban	0	0	1
**Education**	**Frequency**		
Primary	0	1	2
Secondary	17	15	14
Tertiary	3	5	4
**Occupation**	**Unique Responses (frequency)**		
	Farmer (1), Hairdresser (1), Nurse(1), Tailor (1), Teacher (1), Vehicle Insurance Agent (1), Vendor (2), Self-Employed (2), Unemployed (10)	Administrator (1), Hairdresser (1), Teacher (3), Vendor (2), Self-Employed (4), Unemployed (10)	Police Officer (1), Poultry Farmer (1), Tailor (2), Teacher(2), Vendor (1), Self-Employed (5), Unemployed (8)
	**Range**		
**Number of Children**	2 to 6	2 to 7	2 to 5
	**Frequency**		
**Prior Preterm or Low Birthweight Delivery**	7	3	4

**Table 3:** Summary of focus group discussion participants

	Number of Participants	Position at Hospital	Level of Training	Specialty	Total Years of Experience (Sum of Individuals)	Previous Hospital Workplaces
**FGD1**	8	Nurse, Sister in Charge, Matron	Diploma in Midwifery, Diploma in General Nursing	General Nursing, Midwifery, Adminstration	116	Kwekwe General Hospital, Chinhoyi Provincial Hospital
**FGD2**	8	Doctor	Junior Resident Medical Officer, Senior Resident Medical Officer	Obstetrics and Gynaecology, Internal Medicine	14	
**FGD3**	8	Nurse, Sister in Charge	Diploma in Midwifery, Diploma in General Nursing	General Nursing, Midwifery	122	Gweru Hospital, Chinhoyi Provincial Hospital, Parirenyatwa Group of Hospitals, Kadoma/Bindura Hospital
**FGD4**	8	Doctor	Junior Resident Medical Officer	Paediatrics	8	

### Overview of major themes

After analyzing each of the interview types (CS1, CS2, CS3) and the FGDs as distinct entities, it was apparent that information pertaining to several broad topics overlapped across all data types, either from direct responses to questions or organic discussions during an interview or FGD. Of these topics, those most pertinent to maternal and neonatal health and the upcoming emollient clinical trial at SMCH are discussed below: neonatal skincare and bathing at birthing facilities, skincare and bathing at home, beliefs and customs around skincare and the use of oil, neonatal care for preterm infants, the experience of mothers in the NNU, receptivity to an emollient therapy clinical trial, and considerations for a future trial. Information presented in each of the following sections is a synthesis of data from CS1, CS2, and CS3 and the FGDs.

### Neonatal skincare and bathing at birthing facilities

The infant’s first bath was almost universally delayed beyond 24 h. Most mothers reported wiping or drying the baby at the hospital and waiting to bathe the infant until they returned home, with the median hospital stay among women who delivered normal-term infants being 3 days. Mothers used a dry towel, wet towel, soap, “*wipers*” (baby wipes), cotton, or aqueous cream to clean their babies at the hospital, sometimes with the help of nurses.

Nearly all respondents expressed knowledge of placing the newborn on the mother’s bare chest, though most reported not practicing this while at the hospital. Of the five mothers who responded that they did perform skin-to-skin care, two described continuous placement of the newborn (one was a preterm infant) on the chest (as in KMC) for at least one week, while the other three (including one mother of a preterm infant) explained that contact only lasted several minutes. One mother who performed skin-to-skin contact briefly gave the reason of improving infant attachment. Because these responses do not accord with the nurses’ and doctors’ accounts of standard practice, which is to deliver the baby onto the mother’s abdomen, it is likely that respondents here were referring to additional skin-to-skin care which occurred beyond the delivery period. While not asked directly about cord care, many described practicing it at the hospital with cotton and [methylated] spirit.

Focus group discussions with nurses and doctors offered further insight into the standard care for infants after delivery at SMCH. Delivery procedures were described to include all essential aspects of neonatal care: *“As soon as the baby is born, it’s put on mother’s, the mother’s belly um, then the the drying of the baby whilst still on the belly, then the clamping of the cord and the cutting of the cord.”* This is followed by a full examination of the baby, wrapping the baby, encouraging breastfeeding within one hour if the baby is well, and teaching the mother how to perform proper cord care. Drying of the baby was termed “*top and tail*” by nurses. Though not always cited, maternal sentiment towards nurse involvement was positive whenever mentioned by CS1 and CS2 respondents.

Emollient application after drying was practiced by most mothers of normal-term infants while still at the hospital. Mothers whose infants were being cared for in the NNU at SMCH were prevented from applying emollient by nurses and doctors to avoid any risk of burns from phototherapy or incubators. The most popular substance was Vaseline (a petroleum jelly), with alternative commercial products such as Johnson and Johnson’s baby oil and baby gel mentioned. Though nurses initiated the application of substances to the skin among some facility-born infants, in most cases it was the mother who initially began applying the emollient in the hospital.

### Skincare and bathing at home

In alignment with the time mothers spent at the hospital, the median timing of the first bath was after 3 days, since nearly all participants gave their infant the first bath at home, after discharge. Family involvement in bathing—particularly the infant’s maternal grandmother—was cited as centrally important. Most participants reported that the vernix was rubbed off during the first bath.

A small percentage of mothers expressed knowledge of the benefits of delayed bathing, suggesting that mothers may learn about it from their communities or healthcare providers: *“I would dry the baby since the baby is still too young. It’s not good for the baby to be bathed frequently.”*

Another mother delayed bathing a child she suspected to be sick: *“I delayed bathing him and I bathed him after one week. The baby was feeling cold and was coughing. I wanted to bath him, but because he was born with flu, I was not happy. I had no option. But since I delivered a baby with flu, I did not bath him.”*

Many mothers, however, desired to bathe their infants sooner, viewing their infant as “dirty” at birth. When asked their feelings about how the first bath occurred, many expressed a similar sentiment: *“I feel free and happy, because it cleans the baby’s skin and the oil that the baby is born with. So it’s good for me. Baby skin will be left clean and free.”*

Primary bathing substances included: soap (Johnson’s Baby Soap was popular), Aqueous Cream (the brand name Plus Five Aqueous Cream was popular), Lifebuoy, water only, Vaseline, Protex soap, and green bar soap. Bathing occurred 1–2 times per day.

For emollient application after bathing, Vaseline Blue Seal was overwhelmingly the topical emollient applied. Other popular substances included alternative brand-name commercial creams: Baby Max, Epimax, Aqueous Cream, Johnson and Johnson products (oil, gel, cream and soap), Ingram’s Triple Glycerine Cream, and camphor cream. Baby powder was also used. Methods of application almost universally included application to the whole body after bathing and after changing the diaper. The frequency of emollient application was consistent at 2–3 times per day.

One important aspect of skincare among respondents was the application of substances to specific areas of the body. Most common was Vaseline in the diaper area, also described as the “*thighs*,” “*bums*,” and “*nappy area*.” Reasons for this practice mentioned consistently were “*so that they don’t crack because of urine*” and preventing diaper rash. Another common practice was cooking oil application to the fontanelle to prevent dandruff or drying. Singly mentioned areas of application were the armpits, “*hidden areas*,” and the hair.

Besides applying cooking oil to the fontanelle, the use of natural/vegetable oils on infants was only sparsely mentioned. “*Anointing oil*” given by religious organizations and coconut oil were each referenced once. When asked for their opinion on sunflower seed oil in the context of a clinical trial, only one mother conceded: “*most, most of the people, we grew up applying this flower and sunflower oil*.”

The practice of intentionally massaging newborn infants beyond the application of emollient was low among respondents, indicating the practice may not be widespread in the community. Those who did perform massage gave reasons including that it helps the baby relax or sleep, relaxes the joints, or relieves constipation.

### Beliefs and customs related to skincare and oil

Responses uniformly indicated that newborn bathing and skincare are important to respondents’ communities and families. Mothers gain information about this care primarily from family members and friends, though some also cited advice from healthcare professionals. A predominant theme was the centrality of Vaseline to Zimbabwean neonatal skincare. Dubbed by one mother, *“the traditional Zimbabwe petroleum jelly,”* this substance was referenced repeatedly in interviews and FGDs as the universally accepted emollient for newborn skin.

When asked why they chose to use this substance, mothers almost always responded that they were raised with this custom: *“When we grew up we just knew that the baby - you would use Vaseline to apply to the baby’s skin”;* that they observed it in the general practices of those around them: “*Vaseline is encouraged in Zimbabwe for the kids, especially the petroleum jelly”;* and that the wisdom to use Vaseline was passed down generationally: *“Aaah, elders, our parents, we grew up knowing that Vaseline is good for children.”*

Beliefs surrounding skincare were also consistent. The predominant reason for emollient application was to protect skin quality *(“make the baby’s skin smooth,” “make the skin look right,” and “prevent cracks”*). Another common theme was the recognition of the interaction between skincare and weather. Vaseline was described as both providing warmth and protecting infants from the cold, when wind is heavy or during the winter. One respondent used petroleum jelly on an infant’s face as a sunscreen. Less popular, but also cited a few times as reasons for application, were to prevent colds and disease and to help the baby sleep.

Another common Zimbabwean practice mentioned by half of the interviewed mothers was giving infants cooking oil (either boiled or regular) to drink; even those who chose not to give their infants cooking oil were well-aware of the custom. In all cases, cooking oil was given to infants to “*loosen the abdomen*,” to help with constipation, relieve colic abdominal pains, and prevent *“the fontanelle from sinking.”* Many mothers who did not administer cooking oil to their babies explained that doctors had warned against it, or that they used colic drops or gripe water instead for the same purpose.

Because our research team included nurses, one primary concern was respondents’ reluctance to disclose the use of traditional skincare substances typically discouraged at the hospital. Even when asked about their knowledge of what others—not themselves—apply to their infants, participants’ responses focused largely on the commercial substances mentioned above. Nurses and doctors, however, were much more descriptive of traditional skincare practices. From their accounts, cooking oil seemed to be popular, particularly among parents who could not afford commercial baby products. Doctors and nurses also detailed specific instances when they thought that paraffin and religious anointing oil burned infants’ skin, after being applied directly or when infants were placed in incubators, respectively. They conveyed that banana peel ashes and cow dung are at times used for cord care, and often lead to necrotizing fasciitis and other infections, and the mother’s lochia, when rubbed on the skin, can result in cases of sepsis. One nurse also described, “*black substances, different medicines, which they mix, and it becomes thick and black, like she said. Then they apply on the fontanelle. They say they will, to cure the fontanelle, not to fall inside.’”*.

The most referenced “traditional” substance applied to newborn skin across focus groups and individual interviews was a mixture of cooking oil, salt, lemon, and/or ashes (or a subset of these). This, when applied to the bottom of the feet, the whole body, or the fontanelle, was cited to ward off evil spirits, to prevent the fontanelle from sinking, to “*drain secretions*,” and to protect against colds. Also commonly referenced were substances described as specific to the rural areas: soot, snuff (finely ground tobacco), and mutis, homemade mixtures consisting of the crushed leaves from various plants (ruredzo and muzungai).

### Neonatal care for preterm infants

Interviews with mothers whose children were being treated in the NNU and FGDs with nurses and doctors offered insight into care for small and/or sick newborns at SMCH. Though not all mothers had delivered at SMCH (some delivered at home or a nearby clinic), about half of the mothers who gave birth to preterm infants reported practicing skin-to-skin care immediately after delivery. In all cases, preterm neonates were simply dried (no bathing or wiping with water) before receiving immediate medical attention. If admitted to the preterm ward with a birthweight < 1.5 kg, infants were nursed in incubators. Additional treatment includes respiratory support, medications, phototherapy and administration of intravenous fluids and expressed breast milk through oro/nasogastric tube as needed.

Mothers whose infants are in the KMC ward spend their entire day in the ward and practice continuous KMC, only taking a break to go to the bathroom. Infants in that ward are weighed three times a week; Monday, Wednesday and Friday, and are discharged when the baby is able to breast feed, is free from any illness, mother is comfortable to practice KMC at home, and the baby has attained a weight of 1.8 kg.

### Experience of mothers in the neonatal unit

As awareness is increasingly drawn to perinatal mood and anxiety disorders among mothers, particularly those who have given birth to preterm infants, it is important for clinical studies to understand and address the experience of mothers whose infants are in intensive care and the burden this poses to their mental health. Of the 20 mothers interviewed in the NNU of SMCH, three lost a child after delivering twins, four reported stressful delivery circumstances including a hospital transfer, and four were treated for their own medical complications—including postpartum haemorrhage and blindness associated with elevated blood pressure. The length of mothers’ hospital stays ranged from 3 to 23 days, during which mothers either passed all their time in the KMC ward or waited in locker-rooms and hostels to return for feeding their infants in the preterm unit. When asked how she could best be supported while at the hospital, one mother responded: *“Medication and also the right place for us to sleep. And also, what else? Some teachings for us to know some of our other ladies, they don’t know anything. They even, they don’t even ask a doctor, ‘How is my child? What can I do?’ They just go there and nurse their child. So even the teachings for us to be equipped. And also the food, ah the food is very poor.”*

When asked their opinions about the amount of time they see their newborns, immediate post-delivery care, and current care, mothers reported overall satisfaction. A total of 18 out of 20 mothers felt they were part of the decision-making process in issues concerning their child, 19 felt that they were an important part of the team caring for their infants, and all 20 responded that they felt their voice was heard at the hospital. Mothers spoke positively of care from doctors and nurses. Still, many mothers described distress and worry over the state of their child’s health, the insertion of cannulas, baby’s resistance to a feeding tube, and an infant’s stagnant or declining weight. *“It’s so difficult for me to stay far from your child, you go there after three hours, you don’t know what will happen there. Sometimes you go there seeing the oxygen tube removed. It will irritate him. So it might remove the oxygen or the drips, they will be removed. And also, some sisters, maybe they will be busy concentrating on other children because there’s, there’s a lot of children in their ward, about the twenty-three? Yeah. So we are more, we are many. So the care is very little. So when you are out, you think, ‘So? How is my child now? Is he not removing the the oxygen? Am I going to see him alive?’ So you’ll be frustrated.”*

Among all four focus groups, doctors and nurses also conceded a noticeable difference in the mental state of mothers with normal-term versus preterm deliveries:

*Usually you see them seated, seated isolated. You can easily see that they’re in deep. They are sunk in their deep thoughts. When you ask them, they’ll tell you that, ‘Ahh’--they call us mbuyas–‘Mbuya will my, I ever hold my child, mbuya? And then what will happen, mbuya? Mbuya when I go there, mbuya, and see other mothers coming out, I get so devastated. Mbuya, can I. . how am I going to carry on like this? For how long am I going to be in here?’ They’ve got so many questions. And they also need attention from us. They need time for us to talk to them. Yeah. Well, they, they sound like they are so lonely at times.*

When asked what role they wished to take in their child’s care, mothers’ responses included, in order of frequency, to continue following the doctors’ and nurses’ instructions, to be taught how to care for their infants outside the hospital, and to bathe and breastfeed (rather than use a feeding tube or expressing milk into a cup). Respondents widely cited help paying for and finding medicines prescribed by doctors as their predominant need while still at the hospital.

*“We go to the pharmacy and buy the the prescription that we have been written. Every day we will see a new prescription, every day. If you failed to buy them, your child will not be attended. Lastly, we just hear your child has passed away, but you’re wishing to keep him. But because of the lack of money?”*

### Receptivity to an emollient therapy clinical trial

The final component of this study consisted of questions in CS3 for mothers with preterm infants admitted in the NNU and FGDs among nurses and doctors in preparation for a future emollient therapy clinical trial at SMCH. All interview respondents and focus group participants were given a brief explanation of emollient therapy, clinical trials conducted in other countries, and the randomized controlled trial planned for SMCH. Any participant questions were answered by the research team, who were well informed on the future clinical trial protocol.

Overall, reception of the upcoming trial was largely positive: “*Because you’ll be with your child. Maybe it will take less time than staying here in the hospital. Here in the hospital they will create many diseases. They end up saying, ‘Your child is suffering from yellow’. What do they call it? Jaundice? Yes. ‘You need money to go for tests, to take that jaundice to prove is it positive or negative for jaundice?’ So some will be failing because of lack of money. So I think that cosmetic will help us.”*

Of 20 respondents, only three were skeptical of enrollment into the clinical trial, though two stated that they would prefer their infants to be amongst the second group of participants (the control group): *“I want my child to be the second group not the first group. I want to see what will have happened to others.”* All other respondents displayed enthusiasm towards a trial, with their reasonings, in order of popularity, centered around improving their children’s health and chances of survival, scientific evidence supporting the trial, maintaining smooth and healthy skin for their children, and because doctors have recommended it.

All but one respondent stated that if the trial at SMCH showed emollient therapy to be effective, they would continue the practice at home. The two most-cited barriers to emollient use beyond the hospital were cost (14 of 20 participants) and the father’s decision. *“The first thing before I get them, I want to know how I should go about it. Are they affordable? Will they not be expensive for us?” “If the father agrees, yes. If the father agrees, its fine. But if it’s me, the baby can use”.*

When asked their opinion about the age up to which an emollient should be applied at home, most mothers responded not with a chronological measurement but with the opinion that infants should continue to receive applications until they become less susceptible to morbidity and mortality.

Healthcare professionals were also largely receptive to the idea of the clinical trial. Their major concerns were babies suffering from burns in the incubators or during phototherapy, the infants being slipped or dropped by mothers after the oil is applied, and the interruption of normal care (insertion of cannulas, examinations) when the oil is applied. Though both healthcare professionals and mothers were concerned about infants’ skin reacting to the oil, this apprehension was resolved when told that no adverse reactions were reported in previous clinical trials.

### Important considerations for a future clinical trial

FGDs and semi-structured interviews together highlighted several important considerations for a future emollient trial at SMCH and other locations. Eight of 20 mothers responded that they would be upset if assigned to the control rather than the treatment group: *“I will not be happy, because I wish that all the babies will not be applied, or if they want to apply, they should apply all the babies. If you apply, just apply all.”*

In all FGDs, healthcare professionals concurred that mothers in the treatment group would attempt to share the emollient with their control-group counterparts if given the chance. Because nurses and doctors had witnessed mothers’ sharing of medication with each other, they advised that a plan for keeping the oil securely stored would be critical. In the words of one doctor:*“They share most of the things: cotton, spirit, even caffeine citrate, they share between the babies. If one mother has bought it and the other has not bought it yet they share these things.”*

Another physician confirmed this with his experience in the KMC ward.*“I’ve had a case in in KMC. So, so there were two babies, different doses of caffeine. And the mom was trying to advise the other mom that the dose is ‘such and such,’ not knowing that they’re calculated according to the weight of the baby. So you have to be aware of that. So they might share how to, they might share um, even the you know, oil or whatever. So they can share. They will share. They will.”*

Another important idea for future trials to consider is engaging mothers in the oil application. Eight of 20 mothers replied that they would be unhappy if unable to apply the oil to their infants themselves.

*“It pains me. I want to work together with the nurses.”*

*“Mmm, it will be unfair. What if they teach us how to apply the oil”?*

Another mother, when asked, bluntly stated: “*Well, I would do it myself.”*

Four more stated that they would accept the nurse applying the oil but wanted to participate in applying it as well: *“It will pain me, I want to apply myself. But if it’s not allowed and if it’s part of treatment of the child. I will just agree.”*

Consistent feedback among doctors and nurses also supported the involvement of mothers, with healthcare professionals believing it would improve mother-infant bonding, help relieve nurses who may be understaffed, and provide the mothers with a greater sense of agency in their child’s care.

A final important consideration mentioned among one focus group of doctors is the entwinement of emollient therapy with the already-common tradition among some of mixing and applying unknown oils or substances to their infants. These doctors expressed the concern that if proven successful, emollient therapy with sunflower seed oil may potentially invite opportunities for counterfeit oils to be produced and sold informally. It may also open the door for religious and traditional groups who already suggest the application of unknown oils to further experimentation with potentially harmful substances: “*I’m just thinking there’s a possibility that those who are not medical practitioners can jump on the bandwagon and end up having this ‘oil anointing thing’ going on outside the hospital. Unproven, untested things.”*

## Discussion

This study documents critical elements of neonatal care, previously absent from literature, following facility births in Zimbabwe’s capital city. Data reveal adherence to World Health Organization standards of care for the promotion of neonatal health [24], particularly at SMCH—the country’s foremost public-sector tertiary care hospital—where drying and wrapping, skin-to-skin care, delaying the first bath by 24 h, hygienic cord care, and early initiation of breastfeeding are part of routine post-delivery protocol. The study also captures standard neonatal skincare and bathing practices among Harare’s urban population, which center around waiting to bathe at home with the help of family members and applying petroleum jelly or other commercial emollients with an aim to protect the skin, provide warmth, and prevent infection. This formative research indicates that emollient therapy—a cost-effective treatment with the potential to significantly improve preterm neonate outcomes—will likely be received positively by communities in and around Harare because of its alignment with current practices, perceptions, and the needs of new mothers at SMCH. However, it also highlights important challenges and considerations for the trial specific to the site population. These include the opportunity for mothers to help administer the intervention while avoiding spillover of the intervention to the control infants, ensuring the emollient is safely and properly applied in and outside the hospital, and guarding against the use of untested, potentially harmful substances.

This is the first study to examine neonatal skincare, bathing, and emollient use in Zimbabwe. Information on these topics from SSA remains scarce [21, 25]. Besides quantitative data on early breastfeeding from the Demographic and Health Surveys (DHS) in thirty-three countries [26] and qualitative studies on the beliefs and practices surrounding newborn care in rural areas or home settings [27–29], , little qualitative data about facility practices, reasons for facility practices, or the experience of mothers giving birth in a facility currently exists [21, 25, 30]. 

This research adds to evidence from SSA that the prevalence of immediate newborn care practices across and within countries is highly variable. Results contrast with those from most other countries in skin-to-skin contact immediately after delivery, which has generally been reported as low [21], both in quantitative and qualitative studies [27, 31–34]. The high rate of delayed bathing among this sample is consistent with qualitative findings from facilities in Malawi and Tanzania [39], but distinct from Ethiopia, Ghana, Mali, and Uganda, where studies report over 50% of newborns were bathed within 6 h of delivery [27, 31–33]—even among facility births in Ethiopia and Ghana [27]. 

The practice of KMC, defined as the care of preterm or low-birth-weight infants in continuous and prolonged (i.e. 8–24 h per day) skin-to-skin contact initiated immediately after birth, with support for exclusive breastfeeding or breast-milk feeding [35], is also sparsely practiced with limited documentation in the region.

While KMC coverage remains low, long-standing WHO guidelines, country-level policies, and advocacy by global organizations have increased KMC implementation and documentation [35]. As an intervention for small and/or sick newborns in low-resource settings, KMC bears similarities to emollient therapy and research on KMC health system intervention strategies provide insight for the future of emollient therapy. Of thirteen studies evaluating interventions in lower-middle- or low-income- country settings (Bangladesh, India, Ethiopia, Ghana, Nepal, Philippines, Uganda), eight reported increased KMC coverage [35]. From these, lessons relevant to emollient therapy include the necessity for high-level leadership engagement (among all levels of government and facility leadership); ward environments which facilitate the intervention through maternal support and counselling; promotion through community engagement; and early identification and referral of LBW infants for intervention [36]. SMCH’s well-established KMC program offers a unique environment with a strong foundation upon which to introduce emollient therapy. As efforts to improve newborn care continue, the interventions’ complementarity at this study site can serve as a model for strengthening care across maternal, newborn and child health programs worldwide [37, 38]. 

Despite variations in the prevalence of newborn care practices, traditions and motivations surrounding skincare appear to be more homogenous across SSA. Though few studies discuss emollient use, the high prevalence of petroleum jelly applications among our sample is similar to findings from Uganda [39], Ethiopia [40], and Zambia [41]. The use of vegetable oils (cooking oil, sunflower seed oil), coconut oil, and traditional substances made from herbs and leaves described in the results is also common throughout several African countries [25, 28, 29, 39, 40]. Though massage was found to be uncommon in this study as in Uganda [39], studies from other countries including Ethiopia, Egypt, South Africa, Tanzania and Zambia describe massage as a regular part of emollient application, with similar reasons for doing so [25, 40–43]. Primary reasons for emollient application across countries include thermal care, the promotion of healthy skin, protection against infection, and warding off evil spirits [25, 28, 39, 41, 44]. Similarly, primary motivations for bathing, including undesirability of an infant’s “dirtiness” after birth and to help the baby sleep, are consistent across most qualitative findings [21, 44]. This conservancy across qualitative research may point to the generalizability of this study’s findings pertaining to certain traditions in newborn care—namely emollient application and massage—and mothers’, nurses’, and doctors’ opinions, beliefs, and feelings about neonatal skincare.

Few studies to date have been conducted about the acceptability of emollient therapy in a community. Findings from Uganda and Bangladesh report similar enthusiasm for the intervention and its alignment with cultural norms [39, 45]. However, concerns from this study, such as the fear of infant burns in incubators or during phototherapy, mothers sharing the oil, and counterfeit substances emerging, are unique, as is the strong suggestion to include mothers in the intervention. Their responses demonstrate the necessity of fully understanding the specific context for any new intervention as well as their consequences in communities beyond the study or trial site.

### Study limitations

Perhaps the strongest limitation of this study was the potential for respondents’ answers to be influenced by social desirability bias, or a reluctance to admit their true feelings and opinions in a hospital setting to nurses on the research team.

A further limitation is the (seeming) absence of rural representation. Though the patient population of SMCH is taken to be representative of urban, peri-urban, and rural settings surrounding the capital city of Harare, nearly all respondents cited urban addresses when asked where they lived. Despite this, several respondents referenced giving birth at rural clinics or aspects of rural life. One explanation is that mothers coming from outside the city where health care facilities are not easily accessible will typically move to the city for delivery purposes and provide that address when asked at the hospital. Future studies must take note of this distinction in order to accurately understand and represent population demographics.

In addition, the number of study participants who completed higher education is significant. Only 5% of study participants had not completed secondary or tertiary education, and 20% of mothers interviewed had completed tertiary education (Table 2). The 2021 UNICEF Multiple Indicator Cluster Survey of Zimbabwe reported an 83% completion rate of lower secondary school in Harare [46]. Though the rate amongst participants is slightly higher than this figure, researchers believe the study sample is representative of the education level among patients at SMCH. A significant number of public servants with tertiary education cannot afford private hospital care and seek care at public care centers such as SMCH.

Both social desirability bias and an underrepresented rural population may explain why maternal responses to the individual interviews seemed to miss some of the substances described by nurses and doctors in focus groups as more traditional. Beyond this inconsistency, however, general consensus across interview and focus group responses suggests that the results from this study are true to the population of interest.

### Study strengths

This study brings to light neonatal care and skincare practices in an urban Zimbabwean setting, previously absent from literature on this topic [21, 25]. The divergence of findings in this study from the few others in SSA demonstrates the need for more robust documentation of such essential practices across SSA’s many diverse countries to implement appropriate interventions. This study additionally adds to extant qualitative literature on this topic with its focus on birthing facilities: the practices, reasons for such practices, and experiences of mothers who delivered at hospitals or clinics. As facility births increase in the region [47], research to understand why immediate newborn care practices do or do not occur and the consequences of such practices will be critical not only for improving standard of care but also utilizing healthcare facilities to introduce new treatments.

This study also highlights the importance (gaining increasing popularity in the literature [48, 49]) of a thorough understanding of the local context prior to the introduction of healthcare interventions, particularly when such interventions are based in cultural traditions. One key takeaway is the necessity of the intervention to be low-cost, as buying medicines for their children and the cost of a future emollient were cited by mothers as the predominant challenges and barriers to care for their infants. Another important finding from the research is the potential for emollient therapy, like KMC, to allow mothers a greater role in their child’s care in the hospital, meeting a desire expressed by many mothers and a need observed by all nurses and doctors in the NNU.

## Conclusion

This study aimed to elucidate newborn postnatal care, skincare, and bathing practices in Harare, Zimbabwe, and receptivity to an upcoming emollient therapy clinical trial among caregivers and healthcare workers at SMCH. Widespread neonatal skincare and bathing practices include performing the first bath at home with family support and applying petroleum jelly or commercial emollients for various reasons. Current practices, perceptions, and the needs of new mothers suggest emollient therapy will be accepted in and around Harare but pose important challenges and considerations for the trial, such as allowing mothers to help administer the emollient, ensuring safe and controlled emollient usage during the trial and outside the hospital, and preventing the production of potentially harmful substances [50]. 

Improving the care of infants in their first days after birth is essential for ensuring not only decreased mortality, but also healthier populations, a higher quality of life, and social and economic development [2]. Even in settings where facility births are prevalent, low-resource community-based interventions are key to reducing neonatal deaths and decreasing disparities in infant and neonatal mortality around the world. To truly be effective, however, such interventions must be implemented with a thorough knowledge and understanding of the local context, including both the current standard of care and the local customs or traditions. As with Zimbabwe, further research on neonatal care in other LMICs will be important for protecting the health of preterm infants and securing better outcomes for every country’s most vulnerable citizens.

## Data Availability

All study data has been provided in this paper.

## Abbreviations

CS1: Caregiver Survey 1
CS2: Caregiver Survey 2
CS3: Caregiver Survey 3
DHS: Demographic and Health Surveys
FGD: focus group discussion
KMC: Kangaroo Mother Care
LBW: Low birthweight
LMICs: low and middle-income countries
NMR: neonatal mortality rate
NNU: neonatal unit
SMCH: Sally Mugabe Central Hospital
SSA: sub-Saharan Africa
VLBW: very low birthweight
